# Interferon-γ, a valuable surrogate marker of *Plasmodium falciparum *pre-erythrocytic stages protective immunity

**DOI:** 10.1186/1475-2875-10-27

**Published:** 2011-02-08

**Authors:** Blanca-Liliana Perlaza, Jean-Pierre Sauzet, Karima Brahimi, Lbachir BenMohamed, Pierre Druilhe

**Affiliations:** 1 Malaria Vaccine Development Laboratory, Pasteur Institute, 25-28 rue du Dr. Roux, 75724 Paris, Cedex 15, France; 2 Laboratory of Cellular and Molecular Immunology, University of California Irvine, College of Medicine, Irvine, CA 92697-4375, USA

## Abstract

Immunity against the pre-erythrocytic stages of malaria is the most promising, as it is strong and fully sterilizing. Yet, the underlying immune effectors against the human *Plasmodium falciparum *pre-erythrocytic stages remain surprisingly poorly known and have been little explored, which in turn prevents any rational vaccine progress. Evidence that has been gathered in vitro and in vivo, in higher primates and in humans, is reviewed here, emphasizing the significant role of IFN-γ, either as a critical immune mediator or at least as a valuable surrogate marker of protection. One may hope that these results will trigger investigations in volunteers immunized either by optimally irradiated or over-irradiated sporozoites, to quickly delineate better surrogates of protection, which are essential for the development of a successful malaria vaccine.

## Background

Immunity to the pre-erythrocytic stages of malaria (i.e. sporozoite and liver stages), which can be best induced following immunization with radiation-attenuated parasites at sporozoite stage, is unusual in many respects [1-3]. Indeed, it is a very strong, fully sterilizing immunity, and this stands in contrast to immunity to other stages of *Plasmodium*. Since this immunity specifically targets the asymptomatic pre-erythrocytic stage, which precedes the pathogenic blood stages, it can ensure a full prophylaxis suitable for travellers and has, therefore, attracted the vast majority of malaria vaccine funding.

Yet, the immune effectors underlying such a strong pre-erythrocytic immunity, as well as the target sporozoite and liver stage (LS) antigens, remain surprisingly poorly known, at least in the case of *Plasmodium falciparum *in humans [4,5]. Thus, the paradox is that the most promising and effective vaccine against malaria relies on mechanisms which are not identified, nor even searched for. The lack of an immunological basis for protection against *P. falciparum *pre-erythrocytic stages is, in turn, inhibiting any rational progress with the few vaccine candidates under development, and the identification of better candidates [6].

Conversely, over the past 30 years there have been extensive studies conducted using rodent malaria species in laboratory models. These studies have, in a way, added to the confusion by demonstrating a plethora of very effective mechanisms, ranging from sporozoite-neutralizing antibodies to cell-mediated immunity and its various effectors, though without providing a single clue as to which of them would be critical against *P. falciparum *[7].

The difficulties are related to the diversity of the models employed, their relevance and the strong efficacy of each immune effector investigated in one or the other model [8]. Indeed, numerous studies have demonstrated a role for antibody responses in protection, both under *in vitro *and under *in vivo *conditions. In this case, many antibody-mediated mechanisms have been demonstrated including detachment of the sporozoite surface ("circumsporozoite precipitation") [9], opsonization [10], ADCC [11], inhibition of sporozoite invasion and inhibition of intra-hepatic development [12,13]. Conversely, MHC expression by the host cell, the hepatocyte, has understandably led to analysis and description of the potent effects of various T-cell subsets [14-18]. Studies, both *in vitro *and *in vivo*, have established by selective depletion [19-22], knock-outs or adoptive transfer [16,23-26], the role of mainly CD8 cells but also CD4 T-cells (reviewed in [27]), γδ T-cells [28], NK [29,30] and NKT-cells [31], or stellate (Ito) cells[32], thought to act either directly (e.g. by a CTL effect) or indirectly either by their mediators or T-cell driven recruitment of inflammatory cells, predominantly polymorphs macrophages and Kuppfer cells. Among the many immune mediators in which a protective effect was reported, the most potent are interleukin 12 (IL12) [33], interferon gamma (IFN-γ) [20,34-36], O_2_- [37], and NO radicals, but also, NO synthase [38-40], TNF [41,42], IL1 [35], IL6 [43] leading to the secretion of C-reactive protein [44], hemopexin, α1-anti-trypsin and α2-macroglobulin to name but a few, whose ability to block liver schizogony was reported as being highly effective [45].

The diversity of immune effectors also reflects the diversity of the host-parasite combinations in which they were described. Indeed the combination of each of the rodent Plasmodium with each of the many available inbred mice strains, or the natural host the tree-rat Thamnomys, constitute a plethora of situations, which differ from each other and in which the main effector also differs [7,46,47]. A main bottleneck with those models is to determine which of them, if any, is relevant to the human-*P. falciparum *situation. This essential issue remains unresolved. After over three decades of investigations in animal models, the question: "*what would be the most likely protective immune effector and surrogate marker of protection relevant to the human-P. falciparum situation*" remains open and unresolved. Results from such models are thus at best indicative, and at worst irrelevant, and therefore can be misleading [8,46]. This is demonstrated by the large variety of vaccine formulations that induce those immune-effectors which proved highly effective at protecting rodents, yet failed when evaluated in human clinical trials [48-50].

The immune effectors responsible for protection in humans remain unknown, to the extent of being unable to clarify the simplest dichotomy of whether it is mainly dependant on either "antibody" or on "T-cells", at large (reviewed in [4,5,51]).

Previous work with *P. falciparum *irradiated sporozoites, and *P. falciparum*-derived vaccine formulations, particularly liver stage antigen-3 (LSA3) [52], has consistently pointed to IFN-γ as a marker associated with protection against *P. falciparum *pre-erythrocytic stage. The present paper reviews the few available *in vitro *results and more abundant *in vivo *findings in non-human primate models (according to methods which are described in the references quoted below) that document this matter, and highlight how these findings are consistent with reports in humans.

Given the limitations of the rodent models mentioned above, only observations previously made with *P. falciparum *are mentioned, to highlight more clearly than previous reports the critical role of IFN-γ as either a critical immune mediator or at least a valuable surrogate marker of protection.

### IFN-γ: a critical immune mediator and/or surrogate marker of protective immunity against *P. falciparum *pre-erythrocytic stages

Results supporting a critical role of IFN-γ in protective immunity against *P. falciparum *pre-erythrocytic stages come from studies conducted both *in vitro *and *in vivo*, in chimpanzee, *Aotus *and, to some extent, in humans. Data obtained in higher primates are particularly valuable, as several animals underwent multiple challenges. Since all immunized animals were not protected this situation provided an opportunity to compare responses in protected versus non-protected challenges.

### IFN-γ inhibits *P. falciparum *liver schizogony *in vitro*

*In vitro *studies with *P. falciparum *in primary cultures of human hepatocytes revealed a very potent effect of IFN-γ. Indeed, very low concentrations of IFN-γ were efficient against *P. falciparum *liver forms and moderate concentrations were able to fully block the liver schizogonic development. Concentrations of 1 and 10 IU/ml, achieved respectively 90 and 100% inhibition of *P. falciparum *liver schizogony [35], and up to 40% inhibition was still obtained at a concentration as low as 0.1 IU. Subsequent studies confirmed the strong effect of IFN-γ though with minor differences in the extent of inhibition obtained, possibly related either to the recombinant IFN-γ employed, or to experimental conditions. Thereafter, IFN-γ has been frequently included as a positive control for in vitro drug or sporozoite inhibition studies and results have reproduced the initial findings (unpublished material).

### IFN-γ response is associated with protection in *Aotus *monkeys

**Figure 1:**
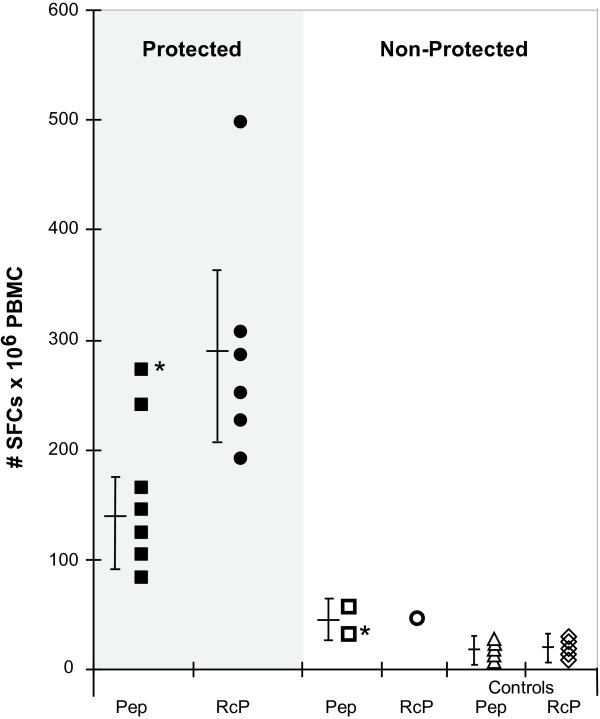
**IFN-γ is associated with protection induced by LSA3 in Aotus monkeys**. Specific IFN-γ responses were studied in nine *Aotus *immunized either by LSA3 recombinant proteins [53] or by long peptides [54]. Monkeys were challenged with *P. falciparum *sporozoites from Santa lucia strain and the IFN-γ responses were evaluated in protected, non-protected and control animals immunized with the GST or ASO2 adjuvant alone. After immunization, the production of IFN-γ was assessed either *ex-vivo by *ELISpot or by ELISA after 40 h of *in vitro *re-stimulation with LSA3 peptides (squares) or Rc proteins (circles), as described in detail in [53] and [55].

In a preliminary study, sterile protection against *P. falciparum *sporozoites challenge (10^5 ^sporozoites IV) was achieved in three out of five monkeys immunized with a microparticulate vaccine formulation of LSA3, without adjuvant [53]. Antibodies reacting with the native parasite sporozoite protein were induced at low titers, but were later boosted following sporozoite challenge. The LSA3-specific IFN-γ secretion, measured either by ELISpot or sandwich ELISA assays, was detectable in all LSA3 immunized animals. However, IFN-γ responses were significantly higher in protected versus non-protected monkeys (Figure 1).

In a second study, monkeys were immunized using two long LSA3 synthetic peptides formulated in the ASO_2 _adjuvant [54]. Protection was achieved in all immunized monkeys challenged with 10^5 ^*P. falciparum *sporozoites, in contrast to controls receiving the ASO_2 _adjuvant alone. Protection in monkeys immunized with either one of the two LSA3 peptides was associated, besides antibody responses, with strong IFN-γ secretion specific to the synthetic immunogen as well as to the native protein. Though all animals of the 2^nd ^study were protected, IFN-γ secretion was at levels similar to those measured in protected monkeys from the first study (Figure 1).

### IFN-γ response is associated with protection in chimpanzees

**Figure 2:**
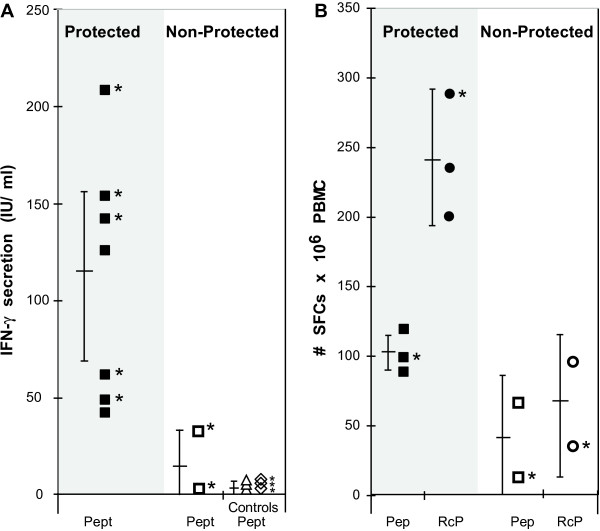
**IFN-γ is associated with protection induced by LSA3 in chimpanzees**. Summary of specific IFN-γ responses in eighteen chimpanzees immunized either by LSA3 recombinant proteins or by Peptides/Lipopeptides. Chimpanzees were challenged once or twice (*) with *P. falciparum *sporozoites. After immunization, the IFN-γ producing T cell responses were compared in protected versus non-protected animals. Chimpanzees that received adjuvant alone or saline alone (vehicle) were used as controls. The production of IFN-γ was assessed after *in vitro *stimulation with LSA3 peptides (squares) or Rc proteins (circles) using either ELISA (A), as described [55] or *ex-vivo *ELISpot (B) as described [53,56].

Three different LSA3 vaccine formulations were assessed in the chimpanzee experimental challenge model. A first group was immunized using a vaccine formulation consisting of LSA3 recombinant proteins delivered in ASO_2 _adjuvant [55]. The antibody responses to different regions of the protein were homogeneously high among both protected and non-protected animals, providing no predictive indication of protection. In contrast, IFN-γ production was markedly higher in the protected chimpanzees (Cyndi and Wendy) compared to non-protected chimpanzees (Marti and Willy) (Figure 2). This was true when considering responses from lymphocytes challenged *in vitro *by either LSA3 recombinant antigens or by synthetic peptides (Figure 2).

A second group of chimpanzees was immunized with a vaccine formulation consisting of two LSA3 lipopeptides, delivered in adjuvant-free saline, and a third LSA3 peptide mixed with montanide adjuvant [55]. Very strong B and T-helper cell responses were observed, and all peptides induced the secretion of IFN-γ, with the highest responses directed to the peptide from the non-repeated N-terminal region. Consistent with results obtained in *Aotus *monkeys, markedly higher IFN-γ levels were observed among chimpanzees showing protection upon challenge (Gerda, Dirk, Mopia, Mgbado), as compared to unprotected animals (Iris, Karlien). The above pattern of immune responses investigated before challenge correlated with protection observed in two successive *P. falciparum *sporozoite challenges performed at either moderate or very high sporozoites challenge dose (20,000 and 1 million sporozoites respectively).

Because the high degree of protection obtained above following LSA3 immunization was not obtained in all animals, it was hypothesized that combination of LSA3 with other pre-erythrocytic stage antigens may act synergistically to result in higher levels of IFN-γ and better protection. Thus, in a subsequent experiment in a third group of chimpanzees LSA3 was delivered in combination with either LSA1, SALSA or STARP. Surprisingly, the combined immunization led to over three-fold decrease in LSA3-specific IFN-γ responses. Indeed, LSA3 alone yielded a mean IFN-γ response of 108 ± 35 IU/ml, whereas the LSA3-specific IFN-γ secretion obtained with the antigen combinations was markedly lower, either with LSA1 (35 ± 21 IU), STARP (16 ± 4 IU) or SALSA (2 ± 0,2 IU). This was associated with an absence of protection as compared to animals receiving LSA3 alone (Thomas A, Millet P *et al*, unpublished material). Hence, here again a relationship was observed between the intensity of IFN-γ response and protection. These results also suggested a negative interaction between antigens, which is noteworthy at a time when indiscriminate antigen combinations are actively promoted.

The difference in IFN-γ levels between protected and non-protected chimpanzees was analysed by a non-parametric median comparison test. The results showed that IFN-γ secretion was significantly higher among protected chimpanzees (p = 0.003). When post-immunization, pre-challenge, IFN-γ responses were analysed with respect to the outcome of all challenges performed in those animals (as some underwent several successive challenges), the correlation between IFN-γ and protection was also significant (p = 0.0002). Conversely, the antibody levels induced in chimpanzee using different immunization protocols were not found to be associated with protection (p = 0.3).

A fourth group of chimpanzees underwent genetic immunization using *P. falciparum *LSA3-DNA [56]. Protection upon challenge was obtained in four out of six immunized animals, and was reproducible upon a 2^nd ^challenge performed five months later. Immune responses induced by DNA vectors were as scarce in chimpanzees as they were in humans, thereby stressing the homology between these two closely related species. For instance, specific antibodies were not detectable. Conversely, positive IFN-γ Elispot responses were obtained in each of the LSA3-DNA immunized chimpanzees, and IFN-γ responses to several peptides was associated with protection, i.e. present in protected animals, and absent in immunized-non-protected ones [56].

In the above studies, responses in chimpanzees were specific to LSA-3 and were composed of both MHC class-I and Class-II restricted T cell responses [55-57]. In liver biopsies, the liver schizonts from immunized chimpanzees were surrounded by a large granuloma containing macrophages, lymphocytes, granulocytes able to release around the parasite, very high local concentrations of IFN-γ, and likely IFN-α [47].

### Indications for the role of IFN-γ in pre-erythrocytic immunity in humans

Surprisingly, only indirect and fragmentary indications of the role of IFN-γ in malaria pre-erythrocytic immunity have been gathered in humans. A first observation is that attenuated *P. falciparum *sporozoites as well as subunit vaccines elicit IFN-γ-producing CD4+ and CD8+ T cells (reviewed in [51,58]). The production of IFN-γ by LSA3 specific T cells was observed in each of three volunteers who were protected following immunization with irradiated sporozoites. However, in contrast with the chimpanzee model, no negative control, similarly immunized but non-protected, is available to assess the predictive value of those IFN-γ responses. More recently, volunteers could be protected by exposure to non-irradiated sporozoites under chloroquine treatment. Strong IFN-γ--producing T cells were observed in all immunized volunteers, whereas antibody responses were inconsistent, supporting the potential role of IFN-γ producing T cells in *P. falciparum *pre-erythrocytic immunity [59]. However, the lack of a control group, similarly immunized, but not protected, prevented the establishment of any causal relationship between the levels of IFN-γ and the observed protection here also.

Correlations of *P. falciparum *antigen specific CD4+ T-cell responses with resistance to malaria in humans have been reported [4]. In studies with CS protein and ME-TRAP vectored vaccines, which induced partial protection (ie. a patency delay) antibody levels induced by this regime were low or absent and were not associated with protection (reviewed in [49]). However, using *ex vivo *and cultured IFN-γ ELISPOT assays, which measure mainly effector and central memory T cells respectively, only the latter showed an association with protection [60,61]. Surrogates were also searched for among the various cohorts immunized by RTSS, showing transient protection. One study identified among individuals without blood parasites for 100 days, a significantly higher frequency of individuals secreting IFN-γ in response to a CS peptide, than of non-responding individuals [62], whereas several other studies found a correlation with antibody responses (reviewed in [4]).

In malaria endemic areas, populations are exposed to a large variety of other infections, particularly of viral origin. It was reasoned that viral infections could induce substantial levels of IFN-γ and this situation could be used as a clue to address the role of IFN-γ on the hepatic cycle in vivo in humans. Indeed, a first study showed high serum levels of IFN-γ, since among 106 individuals tested the mean serum concentration was as high as 70 IU/ml [63]. This subsequently led to the conduction of two immuno-epidemiological studies. The first described a significant correlation between pre-existing serum IFN-γ levels and susceptibility to new malaria blood infections, i.e. the successful transhepatic passage of the parasite [64]. In the second, a very significant difference in time to first infection, i.e. protection, was also observed depending on the ability of patient's lymphocytes to secrete IFN-γ in response to LSA1 antigen [65]. Both observations document the influence of IFN-γ upon the success of exposure to sporozoites in humans. Additional indirect evidence for CD8+ T cell derived IFN-γ comes from the association of the HLA-Bw53 allele with protection [66] as well as class I restricted responses to the LSA3 and PfExp-1 antigens [67]. However, these indications were not confirmed in another location, nor by a parasitological re-assessment of the initial finding [68].

## Discussion

Results gathered in primates challenged by *P. falciparum *and, to some extent, in humans, point to interferon-γ as a possible immune mediator or at least a surrogate marker significantly associated with protection against *P. falciparum *and actually, the only surrogate available to-date.

Despite the high genetic similarity (up to 99.4%) between chimpanzee and humans, the remaining differences limit the scope of these results obtained in these higher primates, with only indirect indications that the same holds true in humans. A second limitation is that most of the results concern immunity induced by LSA3, even though there are indications that the same holds true for humans immunized by irradiated sporozoites, or by other pre-erythrocytic antigen vaccine candidates. Thus data obtained in primates point clearly to IFN-γ as a reliable surrogate of protection, whereas data obtained in humans, in the field or in vaccine trials, is in-keeping with this conclusion, however without demonstrating it as clearly [4,58].

It is extremely striking and shocking that the most critical data, which could have been acquired in human volunteers immunized by irradiated sporozoites, is lacking. This is most regrettable, and surprising, considering the massive amounts of funding devoted to pre-erythrocytic vaccines at large. It does not seem reasonable to continue to evaluate the potential of a large number of vaccine formulations in lengthy and costly phase I and phase II field trials, while the identification of immune effectors or a reliable surrogate marker of protection associated with protection against *P. falciparum *pre-erythrocytic stages is close at hand, yet is not searched for.

Indeed, the opportunity exists for a rational approach in the development of malaria pre-erythrocytic stage vaccines by the identification of the relevant mechanism(s) of protection through clinical experiments with irradiated sporozoite immunization. Complete protection is achieved by vaccination with optimally irradiated sporozoites (15-18 kRad), whereas vaccination with over-irradiated sporozoites (23-30 kRad) induces strong immune responses but no protection [69]. This constitutes a very discriminative situation best fitted to identify either the critical mechanisms involved, or a surrogate marker of protection (such as a certain level of IFN-γ).

There have been to-date more than 1400 volunteers challenged by *P. falciparum *to assess in humans experimental vaccines that were designed in models [70], the majority of which failed to induce protection. In contrast, there have been less than two dozen volunteers immunized by irradiated sporozoites [69]. Arguably the available and unique irradiated sporozoite model has remained largely underexploited for the last two decades, and disconcertingly it still does not seem to attract much interest. Despite the very high failure rate of clinical trials, the current vaccine development strategy continues, by and large, in an empirical manner.

Studies with *P. falciparum *in primates do not bring much support to the role of antibody (Abs) responses in protection against malaria pre-erythrocytic stages, in contrast to what was suggested by numerous experimental studies and which was emphasized in many reviews. The antibody-mediated detachment of the sporozoite surface (circumsporozoite precipitation) and antibody titration in rodents have been considered for a long time as evidence for a major, if not sole, role of antibodies in protection [9]. Further work conducted *in vitro *in primary hepatocyte cultures has indicated a strong sporozoite-invasion inhibitory effect by antibodies both for rodent and for human malaria. This was shown to be true for Abs specific to a wide range of antigens such as CS [71,72], STARP [73], LSA3 [74], SALSA (Pasqueto, V. and Druilhe, P. unpublished data), TRAP [75], AMA-1 [76] as well as when using African adults sera which contain a wide range of antibody specificities [13]. Sporozoite invasion inhibition was also confirmed by *in vivo *passive transfer experiments [77,78]. In addition it was also shown that Abs could promote the opsonization of sporozoites by macrophages [10]. Yet, despite the wide range of evidences in various models, results from *P. falciparum *challenges do not bring support for a critical role of Abs as an effector or a surrogate.

In contrast, for IFN-γ, data gathered using rodent Plasmodium species, despite the limitations of models and the diversity of protective mechanisms involved, are nonetheless in keeping with data obtained with *P. falciparum*. The strong effect of IFN-γ upon liver schizogony was documented in all rodent plasmodia species employed (*Plasmodium berghei, Plasmodium yoelii, Plasmodium chabaudi *and *Plasmodium vinckei*) in various strains of mice [20,27,34,36,79]. The studies conducted in models stressed the effect of each component of the cascade going from IL12 to nitric oxyde. Indeed the potent effect of IL12 [33] is logical as it leads nonT and nonB cells to secrete IFN-γ. The role of IFN-γ itself is the most documented by numerous independent studies [20,34,35], including using IFN-γ receptor knockout animals, which failed to be protected by irradiated sporozoites [36]. The involvement of NO synthase, downstream of the IFN-γ signalling, has been well documented, as well as the direct effect of NO- radicals [33,38,79,80]. Thus studies with rodent species led to dissect this pathway as essential in defence against malaria liver stage (LS), regardless of the target species used.

Observations *in situ *showing a large granuloma around *P. falciparum *liver schizonts in protected chimpanzees [47] have also been analysed and modellized in mice [80]. In those experiments, intra-portal delivery of microparticles coated with *P. falciparum *liver stage peptide epitopes induced strong local cellular infiltrates. This intra-hepatic cellular response detected in mice around the parasite's epitope coated microparticles closely mimics the granuloma detected around chimpanzees' liver schizonts. These immune infiltrates were made up of PMN, MO, dendritic cells, but most importantly of IFN-γ producing activated CD4+ and to a lesser extent CD8+ T-cells, suggesting a Th1 type local immune response [80]. Using different schemes of immunization, the size of intra-hepatic cellular infiltrate was recently found to correlate with the level of protection (Perlaza et al, unpublished data). Indeed, immunization schemes that induced better protective immunity were associated with the strongest local IFN-γ-producing T cell responses. In addition, kinetic studies indicated that, following PMN recruitment, CD4+ T-cells are the first to migrate into the liver toward the parasite antigens. Although in humans the situation is less clear, several indications point more to Th1 CD4+ cells, rather than CD8+ T cells, as the most critical cell type involved in IFN-γ secretion [49]. Production of IFN-γ by PBMC in response to liver stage antigens was also associated with resistance to re-infection with *P. falciparum *in young African children [65]. Vaccine formulations, such as microparticles and lipopeptides, delivered without adjuvant, which proved protective in non-human primates, also induced mainly type-1 CD4+ T cell (Th1) responses but only a low level of CD8+ T cells responses. Finally, in mice, it was previously found that adoptive transfer of parasite-epitope specific Th1 clones, but not Th2 clones, protected mice against a sporozoite challenge [65].

Hence, the combination of data, from both humans and non-human primates, support that protective immunity against *P. falciparum *liver stages is associated with induction of local intra-hepatic helper-type 1 CD4+ T cells that secrete high levels IFN-γ. This suggests that a successful malaria vaccine strategy and formulation should, therefore, promote the induction of strong IFN-γ-producing CD4+ cell responses.

## Conclusions

In conclusion, it was thought to be useful to report these data, whatever their limitations, at least as an incentive to trigger the implementation of further similar investigations in human beings the absence of which is most surprising and appears to be a missed opportunity. Undoubtedly, if there was a protocol able to induce sterile protection against, for instance, HIV infection in humans, as there is with irradiated sporozoites in malaria, this would probably be immediately exploited to analyse the mechanisms and antigens involved. Why this has not and is still not implemented for the pre-erythrocytic stages of malaria remains a mystery. It would seem that limited clinical trials in relatively small groups of volunteers immunized either by optimally irradiated or over-irradiated sporozoites could help delineate surrogate markers of protection, and thereby contribute to the development of a powerful rationale, which is fundamentally required for the development of a successful pre-erythrocytic stage vaccine, and which is currently lacking.

## List of abbreviations

Abs: Antibody; AMA1: Apical Membrane Antigen 1; CS: CircumSporozoite; LS: Liver Stage; LSA1: Liver Stage Antigen 1; LSA3: Liver Sage Antigen 3; Pf Exp1: *Plasmodium falciparum *Export 1; SALSA: Sporozoite and Liver Stage Antigen; STARP: Sporozoite Threonine and Asparagine Rich Protein; Th1: T-helper class1; TRAP: Thrombospondin Related Anonymous Protein.

## Competing interests statement

The authors declare that they have no competing interests.

## Authors' contributions

All authors contributed significantly to the writing of the manuscript. All authors read and approved the final manuscript.
